# The increasing importance of a continence nurse specialist to improve outcomes and save costs of urinary incontinence care: an analysis of future policy scenarios

**DOI:** 10.1186/s12875-018-0714-9

**Published:** 2018-02-17

**Authors:** Margreet G Franken, Isaac Corro Ramos, Jeanine Los, Maiwenn J Al

**Affiliations:** 1 Institute for Medical Technology Assessment, Erasmus University Rotterdam, Rotterdam, The Netherlands; 2 Erasmus School of Health Policy & Management, Erasmus University Rotterdam, Rotterdam, The Netherlands

**Keywords:** Urinary incontinence, Healthcare policy, Community-dwelling elderly, Nurse specialist, Primary care, Future consequences, Cost-effectiveness, Treatment pathways, Ageing population

## Abstract

**Background:**

In an ageing population, it is inevitable to improve the management of care for community-dwelling elderly with incontinence. A previous study showed that implementation of the Optimum Continence Service Specification (OCSS) for urinary incontinence in community-dwelling elderly with four or more chronic diseases results in a reduction of urinary incontinence, an improved quality of life, and lower healthcare and lower societal costs. The aim of this study was to explore future consequences of the OCSS strategy of various healthcare policy scenarios in an ageing population.

**Methods:**

We adapted a previously developed decision analytical model in which the OCSS new care strategy was operationalised as the appointment of a continence nurse specialist located within the general practice in The Netherlands. We used a societal perspective including healthcare costs (healthcare providers, treatment costs, insured containment products, insured home care), and societal costs (informal caregiving, containment products paid out-of-pocket, travelling expenses, home care paid out-of-pocket). All outcomes were computed over a three-year time period using two different base years (2014 and 2030). Settings for future policy scenarios were based on desk-research and expert opinion.

**Results:**

Our results show that implementation of the OSCC new care strategy for urinary incontinence would yield large health gains in community dwelling elderly (2030: 2592–2618 QALYs gained) and large cost-savings in The Netherlands (2030: health care perspective: €32.4 Million - €72.5 Million; societal perspective: €182.0 Million - €250.6 Million). Savings can be generated in different categories which depends on healthcare policy. The uncertainty analyses and extreme case scenarios showed the robustness of the results.

**Conclusions:**

Implementation of the OCSS new care strategy for urinary incontinence results in an improvement in the quality of life of community-dwelling elderly, a reduction of the costs for payers and affected elderly, and a reduction in time invested by carers. Various realistic policy scenarios even forecast larger health gains and cost-savings in the future. More importantly, the longer the implementation is postponed the larger the savings foregone. The future organisation of healthcare affects the category in which the greatest savings will be generated.

**Electronic supplementary material:**

The online version of this article (10.1186/s12875-018-0714-9) contains supplementary material, which is available to authorized users.

## Background

Urinary incontinence is a common, but often neglected, health condition that may impair the quality of life of affected individuals. Reported prevalence rates range from 43 to 77% in nursing home residents [1] and from 4 to 55% in elderly living in the community [2]. Urinary incontinence is often considered as a condition inherent to ageing and many affected individuals are unaware of available treatments [2, 3]. As a consequence, it may take a long time before people tend to seek medical advice; more than half of the affected individuals never seek treatment [4, 5].

Urinary incontinence is often a taboo topic and affected individuals experience feelings of stigma and emotional and social distress [6]. Moreover, urinary incontinence is associated with an increasing risk for falls, fall-related injuries, skin problems, nursing home admissions, and prolonged hospital admissions [7–9] and, consequently, associated with high societal costs [9–11].

Several studies [12–14] have indicated areas for improvement in the management and treatment of urinary incontinence, especially in elderly living in the community. The Optimum Continence Service Specification (OCSS) was developed in order to improve urinary incontinence care in different healthcare settings for four different incontinence patient profiles: i) stress and urgency urinary incontinence, ii) faecal incontinence, iii) neurological induced incontinence, and iv) urinary incontinence in the elderly/ cognitively impaired [15]. The OCSS new care strategy includes active case detection, initial assessment and treatment, improved case coordination, caregiver and community-based support, and use of containment products and technologies [15].

A previous study showed that the implementation of the OCSS new care strategy for urinary incontinence (by the appointment of a nurse specialist in the General Practitioner [GP] practice) in community-dwelling elderly with four or more chronic diseases (i.e. the fourth incontinence profile of the OCSS) resulted in a reduction of urinary incontinence, an improved quality of life, and lower healthcare and lower societal costs in The Netherlands [16]. This implies that postponing the implementation of the OCSS new care strategy costs health and money. Extrapolation of the results of Holtzer-Goor et al. [16] suggest that if the new strategy would not be implemented within the next 15 years, over €145 Million and €585 Million of savings could be forgone in health care and society, respectively. However, mainly due to ageing of the population, it is expected that the number of urinary incontinence affected individuals will rise in the future [17]. Consequently, healthcare and societal costs of continence care most likely further increase, which implies that even greater potential savings could be achievable. Moreover, there is an ongoing trend in (Dutch) healthcare policymaking to shift formal care (covered by insurance) to informal care and to lower the degree of institutionalisation. These demographic and policy changes, in combination with the impact of the condition on the quality of life of patients and the role of informal care givers, make it increasingly important to improve urinary incontinence treatment pathways for community-dwelling elderly. There is, however, no evidence on the future costs and budgetary consequences of continence care.

The objective of this study was to obtain insight into future cost-effectiveness and budgetary consequences of the implementation of the OCSS new care strategy for the treatment of urinary incontinence in elderly with four or more chronic diseases in the primary care setting in The Netherlands and to explore the consequences of various healthcare policy scenarios in an ageing population.

## Methods

We adapted our previously developed decision analytical model [16] to calculate future cost-effectiveness and budgetary consequences of the implementation of the optimum continence service specification (OCSS) for urinary incontinence in the primary care setting in The Netherlands. In line with our previous assessment, we computed all outcomes over a three-year time frame. In this study, however, we used two different base years (2014 and 2030) to enable a comparison of current and future outcomes. Based on expert advice and predicted trends published in the literature, we selected year 2030 as future base year. Further extrapolation would lower the validity and reliability of the results.

### Operationalisation of the optimum continence service specification (OCSS)

The OCSS strategy is not yet implemented in The Netherlands. Therefore, it was operationalised in our model as the appointment of a nurse specialist (at a master level) who is responsible for urinary continence care within the GP practice in the Dutch primary care setting. The implementation of this new care strategy compared to usual care includes the following changes to the current delivery of care: i) more active case detection; ii) initial assessment and treatment by a continence nurse specialist; and iii) improved case coordination [15]. The appointed nurse specialist can either be specialised in continence care visiting several GP practices or specialised in various chronic conditions (e.g., incontinence, stoma and wound care) and appointed within one GP practice. The type of nurse specialist proposed in this evaluation is responsible for urinary continence care and is specially trained to carry out physical examinations, prescribe drugs and containment products, and refer patients to specialist care. Effectiveness estimates of care provided by the nurse specialist in terms of improvement of the condition (mean 21%) and successfully treated (mean 31%) were based on a randomised controlled trial (RCT) by Subak et al. [18]. The international awareness study [19] provided input for the estimations regarding the improvement of case-detection, as this study indicated that the majority of incontinence patients go undetected [16]. Given the lack of evidence, no effects were assigned to improvements related to case coordination.

### Patient population

As in the previous model, the patient population consists of community-dwelling elderly patients (≥65 years) with four or more chronic diseases. The definition of chronic disease was based on an existing list of the International Classification of Primary Care (the ICPC-2 codes of O’Halloran [20] were recoded to ICPC-1 codes used by the Dutch database). The target population was estimated using data from two national databases (Statistics Netherlands and the National Institute for Health Services [NIVEL] Primary Care Database) in combination with incidence and prevalence data from the literature (Teunissen et al. [2] and Uijen et al. [21]). Full details are published in Holtzer-Goor et al. [16]. Table 1 shows the key characteristics of the patient population in the model.

**Table 1:** Key characteristics of the patient population and overview of the settings of the current situation and the 2030 scenarios

Parameter	Current situation (2014)	Scenario 1 (2030)	Scenario 2 (2030)	Scenario 3 (2030)	Scenario 4 (2030)
Number of community-dwelling elderly with ≥4 chronic diseases^a^	860,741	1,420,369 (3.18% annual increase^e^)	1,420,369	1,420,369	1,420,369
Total number of urinary incontinence cases in population (prevalent/ incident)	215,185/ 55,087	355,092/90,904	355,092/90,904	355,092/90,904	355,092/90,904
% of the population annually admitted to a nursing home	4% ^f^	4%	4%	4%	2.95%
Number of patient-years ¥	914,598	1,512,157	1,512,157	1,512,157	1,526,030
Formal home care (covered by insurance)			(annual: 2% reduction of users, and 1% increase of # hours)	(annual: 2% reduction of users, and 1% increase of # hours)	(annual: 1% increase of # of hours)
% users	47%^b^	47%	34.7%	34.7%	47%
# hours per week	6.4 h^g^	6.4 h	7.4 h	7.4 h	7.4 h
Reduction in home care in improved/ success cases	10%/ 25% of number of hours (=0.64/ 1.6 h)^c^	0.64/ 1.6 h	0.64/ 1.6 h	0.64/ 1.6 h	0.64/ 1.6 h
Informal care (time of partner/ family/ friends)			(annual: 2% increase of users and 1% increase of # hours)		(annual: 2% increase of users and 1% increase of # hours)
% users	43%^d^	43%	57.9%	43%	57.9%
# hours per week	12 h ^d^	12 h	13.9 h	12 h	13.9 h
Reduction in informal care in improved/ success cases	10%/ 25% of number of hours (=1.2/ 3 h)^c^	1.2/ 3 h	1.2/ 3 h	0.8/ 2 h	0.8/ 2 h
Private home care (paid out-of-pocket by elderly)					
% users	n/a	n/a	n/a	15.6%	15.6%
# hours per week	n/a	n/a	n/a	4.2 h	4.2 h
Reduction in private care in improved/ success cases	n/a	n/a	n/a	0.4/ 1 h	0.4/ 1 h

*Sources:* ^*a*^*) The definition of chronic disease was based on ICPC-2 codes which were recoded to ICPC-1 codes* [16]*;* ^*b*^*) Sorbye* et al. [28]*;* ^*c*^*) Assumption;* ^*d*^*) Langa* et al. [29]*;* ^*e*^*) Netherlands Institute for Social Research* [22]*;* ^*f*^*) Holtzer-Goor* et al. [16]*;* ^*g*^*) Eggink* et al. [30]

¥ Total of number of patient-years in the three-year time period in the model (including an annual inflow and outflow [admittance to nursing home or mortality])

### Decision analytical model

The decision analytical model was developed to estimate the incremental cost-effectiveness of the implementation of the OCSS for urinary incontinence in The Netherlands [16]. The model captures the complete pathway of Dutch patients as identified by a series of interviews with healthcare experts (3 GPs, 3 pelvic physiotherapists, 2 continence nurses, 3 gynaecologists, 2 surgeons, 2 urologists, a geriatric specialist, a gastroenterologist, and a pharmacists [16]). The pathway included a detection phase (detected and never detected) and a treatment phase (treatment for cure plus containment, treatment for containment only, and self-management). Patients who are never detected remain in this category for the entire duration of the model. Patients who are detected move to the assessment phase and subsequently to the treatment phase. Only patients entering the treatment for cure pathway move into a Markov model consisting of three health states: i) incontinent; ii) improvement; and iii) successfully treated. Full details of the initial decision analytical model (including graphic images of the model) have been published elsewhere [16].

### Input parameters

Most of the model input parameters were identical to the previous study. All were obtained from the literature and national databases, or, in case no data was available, based on expert opinion. Because no patient data was used, ethical approval was not required. Full details are published elsewhere [16]. This paragraphs only describes the adaptations to the previous model. For the current situation (base year 2014), we updated time-dependent model input parameters (i.e., number of community-dwelling elderly with four or more chronic diseases, mortality data, and EURO 2014 prices). We used a two-pronged approach for determining input parameters for forecasting outcomes in 2030. First, a desk-research was performed to retrieve information on trends in healthcare policy and data on demographic developments in The Netherlands, including predictions on ageing, mortality and morbidity of the population. Second, we obtained expert opinion regarding future trends in healthcare policy. In total, three experts participated in the expert panel: one geriatric specialist, one expert from an insurance company involved in healthcare procurement, and one professor in healthcare policy and economics. The first expert meeting focused on the discussion of recent and expected trends in healthcare policy especially regarding the ageing population and the Dutch healthcare system. Based on the first meeting, scenarios were drafted by operationalising future trends in policy. The experts provided written comments on draft scenario settings. In the second expert meeting, we discussed the adapted scenario settings and presented preliminary results. After that, final adaptations were made to the scenario settings. Finally, the experts provided feedback on a draft version of the manuscript.

### Scenarios for forecasting outcomes in 2030

To explore potential consequences of demographic changes in combination with future policy trends, four different scenarios were hypothesised. All scenario settings were based on literature and expert opinion regarding future policy trends. The first scenario only takes into account demographic changes in age, mortality and morbidity of the Dutch population (i.e. an annual increase of 3.18% of the total number of community-dwelling elderly with four or more chronic diseases [22]). The second scenario reflects an ongoing shift from formal home care paid by the health insurer to informal care provided by family and/or friends. An annual reduction of 2% of the numbers of patients using formal home care was assumed in combination with an increase of 1% in the actual number of hours care received (in 2030: 34.7% elderly receive on average 7.4 h formal care per week). This is based on the assumption that persons who no longer receive formal home care are the ones with the lowest need for it. The shift to informal care also results in an annual increase of 2% of informal care receivers alongside an increase of 1% of the number of hours (in 2030: 57.9% of elderly receive on average 13.9 h informal care per week). The third scenario takes into account the expectation that informal caregivers are not able to provide the extra amount of care needed. As a consequence, elderly are forced buying private home care out-of-pocket (unit price €27.80 per hour [23]; in 2030: 15.6% elderly buy 4.2 h per week private home care out-of-pocket). Finally, the fourth scenario reflects a declining trend in institutionalisation of the elderly (in 2030: 2.95%). In case more elderly continue living in the community, they are most likely more severely ill and need, therefore, more care (distributed over formal home care, time investment from informal caregivers, and private home care paid out-of-pocket). In 2030, 47% of elderly receive on average 7.4 h of formal home care, 57.9% of elderly receive on average 13.9 h of informal care, and 15.6% of elderly additionally buy on average 4.2 h of private care out-of-pocket. Table 1 provides an overview of the different scenario settings.

### Statistical analyses and uncertainty analyses

The decision analytical model was developed in MS Excel®; input parameters for the model were adapted for each scenario (see Table 1). Outcomes were calculated for a three-year time frame regarding incremental cost-effectiveness at the patient level and budgetary impact at the national level. Effectiveness estimates were reported as percentage successfully treated and improved patients as well as in Quality Adjusted Life Years (QALYs). The QALY is the most common outcome measure in health economic modelling [24]. One QALY stands for 1 year in perfect health. In case an individual’s health is less than perfect, QALYs are accrued at a lower rate (values can range between 0 and 1). All outcomes were assessed using a healthcare perspective (i.e. costs within the healthcare setting covered within the basic benefit package) and a societal perspective (i.e. healthcare costs and costs related to time investment due to informal care giving, travelling expenses and other out-of-pocket expenses). Parameter uncertainty was assessed as in the initial model [16] using one-way sensitivity analysis (OWSA) by varying each parameter within a range of plus or minus 40% of the mean value. The results of the OWSA were presented in the form of a tornado diagram. Probabilistic sensitivity analysis (PSA) was conducted to assess the uncertainty of parameters simultaneously. Cost parameters were then varied within a range of plus or minus 20% of the mean value. For all parameter values constrained between 0 and 1 (e.g. transition probabilities and utilities), a Beta distribution was applied with a standard error equal to 20% of the mean. A Uniform distribution was assumed for the remaining input parameters. Additional file 1: Table S1 provides details of all input parameters which were included in the PSA, including the base-case settings, probability distribution and their source.

## Results

### Clinical outcomes

Clinical outcomes were consistent in all scenarios because no changes were made to the clinical input parameters. Table 2 shows that the OCSS new care strategy results in better patient outcomes in the incident group as well as in the prevalent group. In the incident group, the difference between usual care and the OCSS new care strategy is 3.8 and 4.0% for successfully treated and improved patients, respectively. In the prevalent group, the difference is 3.4 and 3.1% for successfully treated and improved patients, respectively.

**Table 2:** Clinical outcomes

	Incident group			Prevalent group		
	New care	Usual care	Difference	New care	Usual care	Difference
% success	12.7%	9.0%	3.8%	3.4%	0.0%	3.4%
% improved	11.6%	7.7%	4.0%	3.1%	0.0%	3.1%
% not improved	75.6%	83.4%	−7.7%	93.5%	100.0%	−6.5%

### Results at the individual patient level

Table 3 provides an overview of the results at the individual patient level over a 3-year time period. The table shows that the OCSS new care strategy results in a small QALY gain (0.0051) and is less costly in comparison to usual care in the current and all future scenarios. Total savings per person per 3 year range between €59 [scenario 3] and €316 [scenario 4] in health care and between €355 [scenario 3] and €485 [scenario 4] in society.

**Table 3:** Total quality adjusted life years and costs per person per 3 years

	Current situation (2014)			Scenario 1 (2030)			Scenario 2 (2030)			Scenario 3 (2030)			Scenario 4 (2030)		
	New care	Usual care	Difference	New care	Usual care	Difference	New care	Usual care	Difference	New care	Usual care	Difference	New care	Usual care	Difference
Total QALYs (mean)	2.4829	2.4778	0.0051	2.4829	2.4778	0.0051	2.4829	2.4778	0.0051	2.4829	2.4778	0.0051	2.4829	2.4778	0.0051
Total Costs in EURO (mean)	€33,641	€34,044	-€402	€33,641	€34,044	-€402	€36,655	€37,093	-€438	€33,519	€33,874	-€355	€46,172	€46,657	-€485
Total healthcare costs (mean)	€22,207	€22,303	-€95	€22,207	€22,303	-€96	€19,137	€19,197	-€59	€19,137	€19,197	-€59	€25,674	€25,810	-€136
*General practitioner/Nurse specialist*	€45	€18	€26	€45	€18	€26	€45	€18	€26	€45	€18	€26	€44	€18	€26
*Physiotherapist*	€13	€13	-€1	€13	€13	-€1	€13	€13	-€1	€13	€13	-€1	€12	€13	-€1
*Medical specialist*	€25	€25	€0	€25	€25	€0	€25	€25	€0	€25	€25	€0	€25	€25	€0
*Containment products (insured)*	€570	€441	€128	€570	€441	€128	€570	€441	€128	€570	€441	€128	€569	€441	€128
*UI-related adverse events*	€16	€16	-€1	€16	€16	-€1	€16	€16	-€1	€16	€16	-€1	€16	€16	€0
*Formal home care (insured)*	€21,535	€21,789	-€254	€21,535	€21,789	-€254	€18,465	€18,683	-€218	€18,465	€18,683	-€218	€25,002	€25,297	-€295
*Implementation costs*	€5	€0	€5	€5	€0	€5	€5	€0	€5	€5	€0	€5	€5	€0	€5
Total societal costs (excl. Healthcare costs) (mean)	€11,434	€11,741	-€307	€11,434	€11,741	-€307	€17,518	€17,896	-€379	€14,381	€14,677	-€296	€20,498	€20,847	-€349
*Out-of-pocket expenditures* ^*a*^	€529	€707	-€178	€529	€707	-€178	€529	€707	-€178	€529	€707	-€178	€529	€707	-€78
*Informal care (time family/ friends)*	€10,906	€11,034	-€129	€10,906	€11,034	-€129	€16,989	€17,189	-€200	€10,981	€11,067	-€86	€17,107	€17,240	-€134
*Private home care (paid out-of-pocket)*	–	–	–	–	–	–	–	–	–	€2872	€2903	-€32	€2863	€2900	-€37

^a^Out-of-pocket expenditures consist of costs related to containment products (99.98%) and costs related to travelling expenses (0.02%)

There are only minor differences in costs at the individual patient level between the current situation (2014) and scenario 1 (2030) because the main difference in the model is the total number of patient-years (i.e., differences due to mortality rates are only observable in the decimals). Scenario 2 shows, as expected, a shift from healthcare costs to societal costs because of the shift from formal home care paid by the insurer to informal care provided by family/friends. Because we did not assume a 1:1 substitution ratio (assumption that formal home care covered by the insurer is more efficient), the increase in informal care costs (difference between scenario 1 and 2: new care strategy €6083 and usual care €6155) is higher compared to the decline in costs in formal home care (difference between scenario 1 and 2: new care strategy €3070 and usual care €3106). Scenario 3 shows the results in case informal care is partly replaced by privately out-of-pocket paid home care. Similar, because we did not expect a 1:1 replacement (assumption that people buy fewer hours privately out-of-pocket compared to the number of hours provided by family and/or friends), societal costs are lower compared to scenario 2 (new care strategy: €14,381 vs. €17,518; usual care: €14,677 vs. €17,896). As expected, scenario 4 predicts the highest total costs at the individual patient level for both care strategies (per 3 year: new care strategy €46,172 vs. usual care strategy €46,657). This scenario is the most elaborated scenario regarding resource consumption. However, because the new care strategy is less costly than usual care in all scenarios, scenario 4 predicts the greatest savings from both a healthcare and a societal perspective.

### Budgetary consequences

Table 4 provides an overview of the budgetary consequences. The implementation of the OCSS new care strategy leads in all scenarios to lower costs than usual care, irrespectively of using a healthcare or societal perspective. The greatest savings are, however, incurred by using a societal perspective. It should be noted that the differences between the current situation (2014) and scenario 1 (2030) are driven by the difference in the size of the population due to demographic changes (i.e. total number of patient-years over a 3-year time period). Moreover, the size of the population also slightly increases in scenario 4 (from 1.51 Million to 1.53 Million) because fewer elderly are institutionalised and continue living in the community in this scenario.

**Table 4:** Budgetary impact of implementing the OCSS new care strategy over a period of 3 years

	Current situation (2014)	Scenario 1 (2030)	Scenario 2 (2030)	Scenario 3 (2030)	Scenario 4 (2030)
Total number of patient-years ^a^	914,598	1,512,157	1,512,157	1,512,157	1,526,030
Healthcare perspective	-€ 30.709 M	-€ 50.816 M	-€ 32.410 M	-€ 32.410 M	-€ 72.481 M
Societal perspective	-€ 124.659 M	-€ 206.160 M	-€ 224.231 M	-€ 182.031 M	-€ 250.637 M

*M* million

^a^ Total of number of patient-years in the three-year time period (including an annual inflow and outflow)

**Fig. 1:**
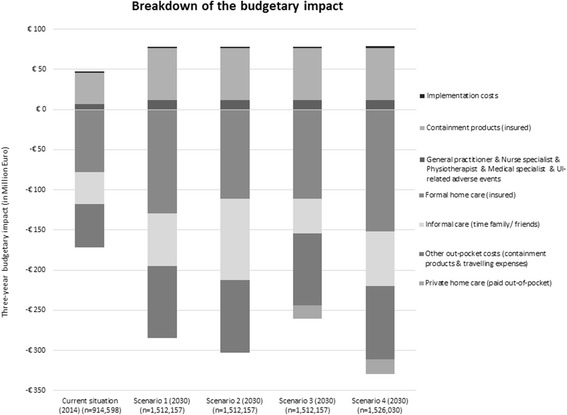
Breakdown of the 3-year budgetary impact

There are differences in which category the greatest costs and cost-savings occur between the scenarios. Figure 1 shows the breakdown of the budgetary impact of the different scenarios (Additional file 2: Table S2 provides full details). The implementation of the OCSS new care strategy results in higher costs in three cost-categories within the healthcare perspective: i) costs of the implementation of the OCSS strategy, ii) treatment costs (including costs for the GP, nurse specialist, physiotherapist, medical specialist, and urinary incontinence related adverse events), and iii) containment products paid by the insurer. The largest costs are attributable to insured containment products. This is mostly due to an increase in the detection-rate of patients with incontinence. The other four cost-categories lead to large savings; one category within the healthcare perspective (formal home care covered through insurance) and three categories within the societal perspective (informal care provided by family/ friends, private home care paid out-of-pocket, and other out-of-pocket expenditures).

The figure illustrates the great impact of different healthcare policies. The shift from formal home care to informal care in scenario 2 and 3 results in lower costs for insured formal home care at the cost of a higher burden for elderly and their family and friends (either in time [scenario 2] or in time and money [scenario 3]). The greatest savings are achieved in scenario 4 because it is the most elaborate scenario on resource consumption. It should be noted that the comparative results of scenario 4 need careful interpretation because of the fact that the economic model only included costs related to urinary incontinence in community-dwelling elderly. Expenditures and/or savings in urinary incontinence affected elderly in other healthcare settings (e.g. elderly living in a nursing home) were not taken into account.

### Uncertainty analyses

**Fig. 2:**
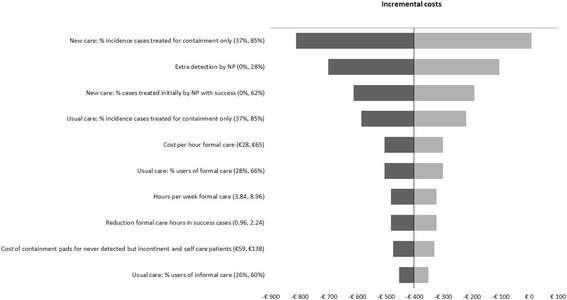
Tornado diagram showing the impact of the ten most influential parameters on the incremental costs for scenario 1

The impact of each input parameter of the model on the incremental costs (per person) was assessed using one-way sensitivity analysis. The tornado diagram (see Fig. 2) shows the ten most influential parameters in scenario 1 (2030). This scenario presents an extrapolation to 2030 of the current situation (2014). In that sense, it represents the base case scenario for future predictions. The ranges of incremental costs are always negative, thus result in lower costs except when the percentage of incidence cases treated for containment only in the new care strategy is increased from 61 to 85%. In this case, the new care strategy could be slightly more costly (€7.87 per patient in 3 years). We observed that parameters associated with the effectiveness of the new care strategy are the most influential on the incremental costs. In particular, we observed that increasing the percentage of incidence cases treated for containment only, the detection rate of urinary incontinence by the nurse specialist, or the success rate of the new care strategy resulted in decreasing costs. Other influential parameters on the incremental costs are associated with the costs of formal home care (unit costs per hour, number of hours provided per week, and reduction of hours in success cases), the frequency of use of formal and informal care in patients treated with usual care, and the out-of-pocket expenditures for containment products incurred by patients managing urinary incontinence with self-care.

The tornado diagrams (not shown) from the other scenarios are similar to scenario 1 (Fig. 2). In fact, the same 10 parameters are most important, but from rank 7 onwards some of the parameters change position.

The results of the propobabilistic sensitivity analysis (PSA; all parameters varied at the same time) are similar for all scenarios. The new care strategy is, on average per person, more effective and less costly compared to usual care. The gain in QALYs ranges from 0.0045 (current situation and scenario 2) to 0.0048 (scenario 1). The incremental cost savings range from €315 (scenario 3) to €430 (scenario 4). Table 5 also shows the PSA estimates for the probability that the new care strategy is i) more effective, ii) less costly, iii) dominant (i.e., more effective and less costly), and iv) cost-effective at a threshold of €20,000. The least favorable setting for the new care strategy is the one considered in scenario 2 (estimated probabilities: 93.0, 92.3, 91.4, and 92.4%, respectively). Table 5 shows a summary of the PSA results.

**Table 5:** Summary of the PSA results

Model outcome	Current situation (2014)	Scenario 1 (2030)	Scenario 2 (2030)	Scenario 3 (2030)	Scenario 4 (2030)
Incremental QALYs per person (2.5%;97.5% percentiles)	0.0045 (−0.0010;0.0130)	0.0048 (−0.0007;0.0144)	0.0045 (−0.0015;0.0136)	0.0046 (−0.0009;0.0137)	0.0047 (− 0.0009;0.0135)
Incremental costs per person (2.5%;97.5% percentiles)	-€345 (−€989; €76)	-€364 (−€994; €42)	-€373 (−€993; €116)	-€315 (−€893; €61)	-€430 (−€1150; €68)
Probability new care is more effective	93.6%	95.0%	93.0%	93.9%	93.8%
Probability new care is less costly	93.7%	94.8%	92.3%	94.0%	94.6%
Probability new care is dominant^a^	92.7%	93.7%	91.4%	92.8%	93.1%
Probability new care is cost-effective at €20,000/ QALY threshold	93.8%	95.0%	92.4%	94.4%	94.4%

*QALY* quality adjusted life years

^a^ Dominant means that the new care is more effective and cost-saving

Furthermore, the implementation of the OCSS new care strategy remained cost-saving even in extreme case scenarios. For example, halving the reduction in numbers of hours formal home care and informal care in success (reduction from 25 to 12.5%) and improved (from 10 to 5%) patients resulted in almost halving the cost-savings in both categories (budgetary impact formal home care: from €129 Million to €65 Million; informal care: from €65 Million to €33 Million), and almost halving (from €206 Million to €109 Million) the total societal savings (using the settings in scenario 1). Although the implementation of the OCSS new care strategy remained less costly at the individual patient level using a societal perspective (€211 lower costs per patient per 3 year), total costs increased, however, in case of using a healthcare perspective (from €96 lower costs to €31 per patient per 3 year). Another extreme case scenario showed the impact of assigning the reduction in the numbers of hours in success and improved patients to either informal care or private care paid out-of-pocket (using the settings in scenario 3). The former scenario resulted in even greater total cost-savings (i.e. the additional savings in informal care [€22 Million] outweigh the additional costs in home care paid out-of-pocket [€16 Million]). Although savings (€32 Million) in private care paid out-of-pocket tripled in the latter scenario (from €16 Million to €48 Million), this did not outweigh the additional costs in informal care (€44 Million).

## Discussion

We investigated the long-term impact of implementing the OCSS new care strategy for urinary incontinence for community-dwelling elderly with four or more chronic diseases in The Netherlands. A previous study showed that the appointment of a nurse specialist (who improves case detection, treatment effectiveness, and case-coordination) would result in a reduction of urinary incontinence, and thereby would lead to an improved quality of life, lower healthcare, and lower societal costs in community-dwelling multi-morbid elderly in The Netherlands [16]. Because of demographic changes in an ageing population, the number of elderly affected with urinary incontinence will rise in the future [17]. Our future scenarios (in 2030) reveal that these changes only further necessity an appropriate strategy to manage the increasing needs of elderly living in the community. Although we cannot foretell future healthcare policies, our results illustrate that various realistic policy scenarios (defined by experts) lead to large health gains and cost-savings by implementing the OSCC new care strategy for urinary incontinence in 2030 (QALYs: 2592–2618; costs health care perspective: €32.4 -€72.5 Million; costs societal perspective: €182.0–250.6 Million). The different scenarios clearly explicate where the greatest cost-savings can be generated (i.e., healthcare payer, society, or the affected elderly and his/her family). We estimated that there is a high probability (> 92% in all our scenarios) that the OCSS new care strategy is more effective and less costly, irrespectively of healthcare setting. Even in extreme case scenarios our results appeared to be rather robust and the implementation of the OCSS new care strategy remained cost-saving compared to usual care.

Several studies [12–14] indicated areas for improvement in the management and treatment of urinary incontinence. Our results show that improvement of care pathways by implementing a continence nurse specialist not only results in better clinical outcomes but also contributes to important cost-savings. It should be noted that our study only investigated consequences in the community setting. Therefore, some of the savings and/or expenditures in care for community-dwelling elderly with urinary incontinence may be at the cost of spending and/or saving money in urinary incontinence affected elderly in non-community settings. For example, the population in the fourth scenario is slightly different from the first three scenarios (i.e., greater number of patient life-years over a 3-year time period due to a lower rate of institutionalisation [1,526,030 vs. 1,512,157]). Therefore, the results on the budgetary impact between the scenarios need careful interpretation.

The effectiveness estimates of care provided by a nurse specialist in our study are based on an RCT by Subak et al. [18] and on the awareness study [19]. Other studies reported conflicting outcomes regarding the effectiveness and costs of a nurse specialist. These studies are, however, not entirely comparable to our study. Moore et al. [25] reported no differences in health outcomes between conservative treatment provided by continence nurse advisors and urogynaecologists in the United Kingdom (UK). A more recent study in the UK by Williams et al. [26] showed better health outcomes due to the new nurse led service but at higher costs. In contrast to our study, Williams et al. [26] did, however, not include costs of formal home care and informal care. Although in our study costs related to other healthcare resources also rise, savings related to formal home care are much greater. Similarly, a Dutch study by Albers-Heitner et al. [27] did not include costs of formal home care and informal care. This, in combination with the fact that they only used a one-year timeframe (which resulted in higher implementation costs), mainly explains the differences in results. Furthermore, previous results of the model published by Holtzer-Goor et al. [16] showed that the new care strategy still yielded QALY gains and resulted in lower costs even in case the new care strategy was assumed only to impact the detection rate and not the effectiveness rate. Our extreme case scenarios further underline the robustness of our results.

To our knowledge, no other studies investigated future consequences of the introduction of a nurse specialist led service. The expected increase in numbers of community-dwelling elderly with urinary incontinence in combination with the impact of the condition makes it increasingly important to improve urinary incontinence care pathways. Our results are specific for The Netherlands where the GP has an important role in the treatment and coordination of care for community-dwelling elderly with urinary incontinence (the GP acts as a gatekeeper to specialist care). Other countries may have a different organisation of the healthcare system and the primary care setting. Moreover, (future) healthcare policies are context specific. Although beyond the scope of our study, in case the new care strategy will be implemented, the description and details of this nurse led service and its finance mechanism should be further developed based on the components as described by Wagg et al. [15] taking into account context specific healthcare policies and organisation. We believe, however, that our study provides valuable insights for (primary) healthcare settings in other countries.

Our extensive analyses including several realistic policy scenarios illustrate that investing in the improvement of urinary incontinence treatment pathways most likely results in large health gains and large cost-savings, irrespectively of the healthcare setting. Although the average impact at the individual patient level seems relatively small (i.e. QALY gain: 0.005; cost-savings: €59 - €316 in healthcare and €355 - €485 in society), it is important to realise that many patients remain undetected or receive care for containment only in which case no gains were assigned in the model. Thus, the impact in individual patients who were successfully treated is much greater.

To maintain sustainability of the healthcare system, there is an ongoing trend to lower the degree of care covered within the basic benefit package (including lowering the rate of institutionalisation) and to emphasise the expanding role of informal care. This may result in an increase of unmet needs and may put pressure on informal caregivers and/or on personal budgets. The extent to which the consequences put pressure on the elderly and their family may differ by country. However, we believe that other countries experience similar issues as The Netherlands, albeit to a varying degree.

Because future consequences of demographic and healthcare policy changes are not yet clear, it is crucial to carefully evaluate their potential impact and facilitate the increasing needs appropriately. The OCSS strategy is an example how a new care strategy can be implemented to facilitate such changes and, simultaneously, improve the sustainability of the system, irrespectively of the healthcare setting. More importantly, our study reveals that implementation of the OSCC new care strategy is cost-saving from a healthcare as well as from a societal perspective, irrespective of whether it is implemented now or in the future. However, our results also clearly highlight the great urgency to implement such a strategy in the near future. Postponing the implementation implies foregoing large health gains and savings. For example, assuming linearity (i.e., average of the current scenario [2014] and scenario 1 [2030]) would imply foregoing €204 Million savings in health care and €827 Million savings in society in the next 15 years in The Netherlands. Similarly, community-dwelling elderly can gain on average 693 QALYs per year. Thus, this would imply forgoing 10,401 QALYs in the next 15 years. Furthermore, informal care was computed as costs for society, which is best practice in economic evaluations. Informal caregiving is, nevertheless, time spend on caregiving instead of time spend on, for example, work or leisure. Implementation of the OSCC new care strategy would result in 1.27 Million fewer hours of informal caregiving per year (19.09 Million hours in the next 15 years).

Finally, it should be mentioned that there is limited data available for various areas of incontinence care [16]. In The Netherlands, the role of the nurse specialist is quite new and it will take some time before a program like the OCSS can be delivered on a large scale. It is, therefore, of utmost importance to introduce the OCSS in a study setting and carefully monitor all aspects.

## Conclusion

Prevalence and incidence of urinary incontinence will rise in an ageing population which will put pressure on current treatment pathways. It is, therefore, inevitable to improve the management of care for community-dwelling elderly with incontinence. Implementation of the OCSS for urinary incontinence most likely results in a reduction of urinary incontinence, an improvement in the quality of life in community-dwelling elderly, a reduction of the costs for payers and affected elderly, and a reduction in time invested by carers. Although we cannot foretell the future, our study indicates that various realistic policy scenarios forecast even larger savings in the future. More importantly, the longer the implementation of such a program is postponed the larger the health gains and cost-savings foregone. The future organisation of healthcare influences where the greatest savings can be generated.

## Additional files

Additional file 1: Table S1. Input parameters of the model. (DOCX 42 kb)

Additional file 2: Table S2. Breakdown of the budgetary impact over a period of 3 years. (XLSX 12 kb)

## Abbreviations

GP: General practitioner
ICPC: International Classification of Primary Care
NIVEL: Netherlands Institute for Health Services
OCSS: Optimum Continence Service Specification
OWSA: One-way sensitivity analysis
PSA: Probabilistic sensitivity analysis
QALY: Quality adjusted life year
RCT: Randomised controlled trial
UK: United Kingdom
