# Supplementary material for: The increasing importance of a continence nurse specialist to improve outcomes and save costs of urinary incontinence care: an analysis of future policy scenarios
Source: BMC Fam Pract. 2018 Feb 17;19:31. doi: 10.1186/s12875-018-0714-9 (PMC5816541; doi:10.1186/s12875-018-0714-9)
Supplement: Supplementary file 1 — Input parameters of the model. (DOCX 42 kb) [file 12875_2018_714_MOESM1_ESM.docx]

**Table S1. Input parameters of the model**

| **Parameters** | **Base case value** | **Probability distribution** | | **Sources** | |
| --- | --- | --- | --- | --- | --- |
| ***Incidence and prevalence*** | | | | | |
| Prevalence of any UI in ≥65 years of age  with 4+ comorbidities | 25% | Beta(18, 55)**^*^** | | Statistics Netherlands (CBS). Available: <https://opendata.cbs.nl/statline>  Uijen et al. 2008. PMID: 18949641  Teunissen et al. 2004. PMID: 14752592  Holtzer-Goor et al. 2015. PMID: 26426124 | |
| Incidence of any UI in ≥65 years of age  with 4+ comorbidities | 3.2% | Beta(23, 731) **^*^** | | Netherlands institute for health services research (NIVEL). Available: <https://www.nivel.nl/en>  Holtzer-Goor et al. 2015. PMID: 26426124 | |
| ***Transition probabilities for usual care*** | | | | | |
| ***Patient flow prior to treatment*** | | | | | |
| Usual care: % incident cases detected by GP | 50% | Beta(12, 12) **^*^** | | Bacher J. Awareness about incontinence among the general public. Paper presented at the 5ft Global  Forum on Incontinence: Better Care, Better Health-Towards a Framework for Better Continence Solutions.  Madrid. Available: http://www.gfiforum.com/Documents/Madrid-2014/Conference%20Summary  %20Report%205th%20GFI.pdf | |
| Usual care: % incident cases assessed/diagnosed by GP | 95% | Beta(17, 1)^**^ | | Assumption – Holtzer-Goor et al. 2015. PMID: 26426124 | |
| ***Treatment strategy*** | | | | | |
| **Treated for cure** | | | | | |
| Usual care: % prevalent cases treated for cure | 0.01% | Beta(24, 249949) **^*^** | | Derived with model - Holtzer-Goor et al. 2015. PMID: 26426124 | |
| **Treated for containment only** | | | | | |
| Usual care: % incidence cases treated for containment only | 61% | Beta(9, 6) **^*^** | | Expert opinion | |
| Usual care: % prevalent cases treated for containment only | 36% | Beta(15, 28) **^*^** | | Calculated: in incident population 30% is in containment  after 3 years, 83% failures: 0.3/0.83=0.36 | |
| **Not treated - self care** | | | | | |
| Usual care: % incident cases not treated - self care | 2% | Beta(24,1200) **^*^** | | Assumption – Holtzer-Goor et al. 2015. PMID: 26426124 | |
| Usual care: % prevalent cases not treated - self care | 1.2% | Beta(24, 2033) **^*^** | | Calculated: in incident population 1% is in self-care  after 3 years, 83% failures: 0.01/0.83=0.012 | |
| ***1st line treatment action*** | | | | | |
| ***GP*** | | | | | |
| Usual care: % prevalent cases treated by GP | 33% | Beta(16, 33) **^*^** | | Assumption – Holtzer-Goor et al. 2015. PMID: 26426124 | |
| Usual care: % initially referred from GP | 51% | Beta(11, 11) **^*^** | | Expert opinion | |
| Usual care: % referred from GP to physiotherapist | 57% | Beta(10, 8) **^*^** | |  |  |
| Usual care: % receiving meds from GP with improvement | 63% | Beta(8, 5) **^*^** | | Drutz et al. 1999. PMID: 10543335 | |
| Usual care: % receiving meds from GP with success | 16% | Beta(20, 110) ^*^ | | Imamura et al. 2010. PMID: 20738930 - Average % patients 'absolutely dry' after using tolterodine IR, tolterodine ER, solifenacin, darifenacin or fesoterodine. | |
| Usual care: % users of medication that continue | 76% | Beta(5, 2) **^*^** | | Sexton et al. 2011. PMID: 21489081 - Average discontinuation rate from the studies that reported results at 9-12 months for the medications: tolterodine, solifenacin, darifenacin, fesoterodine. | |
| Usual care: % receive lifestyle advice from GP | 17% | Beta(20, 100) **^*^** | | Expert opinion | |
| Usual care: % receiving lifestyle advice from GP with improvement | 10% | Beta(22, 202) **^*^** | | Assumption – Holtzer-Goor et al. 2015. PMID: 26426124 | |
| Usual care: % receive other therapy (treatment of infections) from GP | 4% | Beta(23, 575) **^*^** | | Expert opinion | |
| Usual care: % receive other therapy from GP with improvement | 40% | Beta(14, 22) **^*^** | | Assumption – Holtzer-Goor et al. 2015. PMID: 26426124 | |
| Usual care: % receive other therapy from GP with success | 10% | Beta(22, 202) **^*^** | |  |  |
| ***PT*** | | | | | |
| Usual care: % receive PFMT training only | 95% | Beta(18, 1) **^**^** | | Expert opinion | |
| Usual care: % receiving PFMT with improvement | 62% | Beta(9, 5) **^*^** | |  |  |
| Usual care: % receiving PFMT plus biofeedback with improvement | 45% | Beta(13, 16) **^*^** | | Burns et al. 1993. PMID: 8315230 - Average across all severity groups: % subject with 50-99% fewer episodes. | |
| Usual care: % receiving PFMT plus biofeedback with success | 23% | Beta(18, 64) **^*^** | | Burns et al. 1993. PMID: 8315230 - Average across all severity groups: % subject with 100% fewer episodes. | |
| Usual care: % training patients in the second cycle with improvement | 44% | Beta(13, 18) **^*^** | | Mcfall et al. 2000. PMID: 11067699 - Proportion based on 27% that were improved and assume failure is 0% after initial improvement | |
| Usual care: % PT failures referred to GP | 33% | Beta(16, 33) **^*^** | | Expert opinion | |
| ***2nd line treatment action*** | | | | | |
| Usual care: % referred initially from SP to PT for training | 40% | Beta(14, 22) **^*^** | | Expert opinion | |
| Usual care: % receive surgery from specialist | 17% | Beta(20, 100) **^*^** | | Expert opinion. Only women with pure SUI receive surgery: 90% of SUI are operated and 19% of women have SUI and 80% are women (no info on men, assume the same for men). | |
| Usual care: % receiving surgery from SP with improvement | 8% | Beta(22, 264) **^*^** | | Expert opinion (average improvement rate of ProAct and TVT procedure). | |
| Usual care: % receiving surgery from SP with success | 77% | Beta(5, 2) **^*^** | | Labrie et al. 2013. PMID: 24047061 | |
| Usual care: % receive conservative therapy from specialist | 2% | Beta(24, 1248)^*^ | | Expert opinion: 2.5% of women with UUI or MUI receive conservative treatment and 77% of patients have MUI or UUI and 80% are women (no info on men, assume the same for men). | |
| Usual care: % receiving conservative therapy from SP with improvement | 34% | Beta(15, 31) **^*^** | | Richter et al. 2010. PMID: 20177294 | |
| Usual care: % receiving meds from SP with improvement | 63% | Beta(8, 5) **^*^** | | Drutz et al. 1999. PMID: 10543335 | |
| Usual care: % receiving meds from SP with success | 16% | Beta(20, 110) **^*^** | | National Collaborating Centre for Women’s and Children’s Health & National Institute for Health and  Care Excellence (NICE). Urinary incontinence in women: The management of urinary incontinence in  women. Royal College of Obstetricians and Gynaecologists. 2013. Available: http://www.nice.org.uk/  guidance/CG171.  Based on average % patients 'absolutely dry'  after use of tolterodine IR, tolterodine ER, solifenacin,  darifenacin, or fesoterodine. | |
| Usual care: % users of medication that continue | 76% | Beta(5, 2) **^*^** | | Sexton et al. 2011. PMID: 21489081  Average discontinuation rate from the studies that reported results at 9-12 months for the medications: tolterodine, solifenacin, darifenacin, fesoterodine. | |
| ***Other events*** | | | | | |
| Usual care: Incidence of urinary tract infection | 8% | Beta(22, 281) **^*^** | | Hu and Wagner 2005. PMID: 16086679 | |
| Usual care: Incidence fractures | 0.02% | Beta(24,109598) **^*^** | | Meerding et al. 2006. PMID: 16476683  Incidence of wrist and hip fractures. | |
| Usual care: Incidence skin breakdown | 8% | Beta(22, 264) **^*^** | | Brown et al. 2000. PMID: 11183900  Patients with OAB receiving treatment for skin infections. | |
| ***Use of care outside health care sector*** | | | | | |
| Usual care: % users of informal care | 43% | Beta(13, 18) **^*^** | | Langa et al. 2002. PMID: 11982676 | |
| Usual care: % users of formal care | 47% | Beta(12, 14) **^*^** | | Sorbye et al. 2009. PMID: 18785918 | |
| ***Transition probabilities for new care*** | | | | | |
| ***Patient flow prior to treatment*** | | | | | |
| Extra detection by NP | 14% | Beta(6, 41) **^**^** | | Bacher J. Awareness about incontinence among the general public. Paper presented at the 5ft Global  Forum on Incontinence: Better Care, Better Health-Towards a Framework for Better Continence Solutions.  Madrid. Available: http://www.gfiforum.com/Documents/Madrid-2014/Conference%20Summary  %20Report%205th%20GFI.pdf  Assumption: 14% more than usual care | |
| ***Treatment strategy*** | | | | | |
| **Treated for containment only** | | | | | |
| New care: % incidence cases treated for containment only | 61% | Beta(9, 6) **^*^** | | Expert opinion | |
| New care: % prevalent cases treated for containment only | 44.5% | Beta(13, 17) **^*^** | |  |  |
| ***1st line treatment action*** | | | | | |
| ***GP and NP*** | | | | | |
| New care: % prevalent cases treated by GP/NP | 33% | Beta(16, 33) **^*^** | | Assumption – Holtzer-Goor et al. 2015. PMID: 26426124 | |
| New care: % cases treated initially by NP with improvement | 21% | Beta(2, 12) | | Subak et al. 2002. PMID: 12100806 | |
| New care: % cases treated initially by NP with success | 31% | Beta(6, 14) | |  |  |
| New care: % initially receive other therapy (treatment of infections) | 4% | Beta(23, 575) **^*^** | | Expert opinion | |
| New care: % patients initially receiving other therapy from NP with improvement | 40% | Beta(14, 22) **^*^** | | Assumption – same as in usual care | |
| New care: % patients initially receiving other therapy from NP with success | 10% | Beta(22, 202) **^*^** | |  |  |
| New care: % NP failures continuing with NP care | 40% | Beta(14, 22) **^*^** | | Assumption – Holtzer-Goor et al. 2015. PMID: 26426124 | |
| New care: % failures referred from GP/NP | 60% | Beta(9, 6) **^*^** | |  |  |
| New care: % GP/NP failures referred to PT | 33% | Beta(16, 33) **^*^** | |  |  |
| ***PT*** | | | | | |
| New care: % receive PFMT training only | 95% | Beta(0.3, 0.02) **^*^** | | Expert opinion | |
| New care: % receiving PFMT with improvement | 37% | Beta(15, 26) **^*^** | | Expert opinion.  Adjusted the proportion of improved patients  (from 62% to 37%) because of the initial treatment  by the NP was already successful in a part of the patients | |
| New care: % receiving PFMT plus biofeedback with improvement | 45% | Beta(13, 16) **^*^** | | Burns et al. 1993. PMID: 8315230  Average across all severity groups: % subject with 50-99% fewer episodes. | |
| New care: % receiving PFMT plus biofeedback with success | 23% | Beta(18, 64) **^*^** | | Burns et al. 1993. PMID: 8315230  Average across all severity groups: % subject with 100% fewer episodes. | |
| New care: % training patients in the second cycle with improvement | 46% | Beta(13, 15) **^*^** | | Mcfall et al. 2000. PMID: 11067699  (50-99% dry)  Adjusted the reported proportion of improved patients  (from 27% to 17%) because of the initial treatment  by the NP was already successful in a part of the patients | |
| New care: % PT failures referred to GP | 33% | Beta(16, 33) **^*^** | | Expert opinion | |
| ***Quality of Life*** | | | | | |
| Utility success | 0.860 | Beta(1725, 282) | | Slieker-ten Hove et al. 2010. PMID: 19634171 | |
| Utility improvement | 0.842 | Beta(1861, 349) | |  |  |
| Utility failure | 0.825 | Beta(2568, 546) | |  |  |
| ***Costs^***^*** | | | | | |
| Hours per week formal care | 6.4 | Uniform*^***^* | Expert opinion | |  |
| Cost per hour formal care | €46.68 | Uniform*^***^* | Holtzer-Goor et al. 2015. PMID: 26426124 - Average number of hours per week of personal home care at a price of €42.47. Reimbursement per week. Achmea ZV. Assume 10% reduction. | |  |
| Hours per week informal care | 12.04 | Uniform*^***^* | Expert opinion | |  |
| Cost per hour informal care | €13.74 | Uniform*^***^* | Assumption – Holtzer-Goor et al. 2015. PMID: 26426124  Based on average number of hours per week of personal home care at a price of 42.47. Reimbursement per week. Achmea ZV. Assume 10% reduction. | |  |
| Reduction formal care hours in success cases | 1.60 | Uniform*^***^* | Expert opinion: 25% of the hours per formal care. | |  |
| Reduction formal care hours in improved cases | 0.64 | Uniform*^***^* | Expert opinion: 10% of the hours per formal care. | |  |
| Reduction informal care hours in success cases | 3.01 | Uniform*^***^* | Expert opinion: 25% of the hours per informal care. | |  |
| Reduction informal care hours in improved cases | 1.20 | Uniform*^***^* | Expert opinion: 10% of the hours per informal care. | |  |
| Cost per hour particular care | €27.80 | Uniform*^***^* | Hakkaart-van Roijen L, Tan SS, Bouwmans CAM. (2011) Handleiding voor kostenonderzoek, methoden  en standaard kostprijzen voor economische evaluaties in de gezondheidszorg. geactualiseerde  versie 2010. Diemen: Department FIA CVZ.127p. | |  |
| ***1st line assessment and treatment*** | | | | | |
| ***GP*** | | | | | |
| Consultation cost GP - usual care | €30.78 | Uniform*^***^* | Hakkaart-van Roijen L, Tan SS, Bouwmans CAM. (2011) Handleiding voor kostenonderzoek, methoden  en standaard kostprijzen voor economische evaluaties in de gezondheidszorg. geactualiseerde  versie 2010. Diemen: Department FIA CVZ.127p. | |  |
| Cost of meds - GP | €116.09 | Uniform*^***^* | National Health Care Institute [Zorginstituut Nederland]. Farmacotherapeutisch kompas. Available:  <http://www.farmacotherapeutischkompas.nl/>  Holtzer-Goor et al. 2015. PMID: 26426124 | |  |
| Cost of lifestyle advice - GP | €30.78 | Uniform*^***^* | Hakkaart-van Roijen L, Tan SS, Bouwmans CAM. (2011) Handleiding voor kostenonderzoek, methoden  en standaard kostprijzen voor economische evaluaties in de gezondheidszorg. geactualiseerde  versie 2010. Diemen: Department FIA CVZ.127p. | |  |
| Cost of training - GP | €30.78 | Uniform*^***^* |  |  |  |
| Cost of other (treatment of infections) - GP | €2.53 | Uniform*^***^* | National Health Care Institute [Zorginstituut Nederland]. Farmacotherapeutisch kompas. Available:  <http://www.farmacotherapeutischkompas.nl/>  Holtzer-Goor et al. 2015. PMID: 26426124 | |  |
| ***GP/NP*** | | | | | |
| Consultation cost NP | €41.80 | Uniform*^***^* | Assume the NP spends one hour with each patient in the initial diagnosis/assessment phase.  NVZ (Nederlandse vereniging van ziekenhuizen). Available: http://www.nvz-ziekenhuizen.nl/_library/  398/7.1.1 Salarisschalen per1-7-2012.pdf  Holtzer-Goor et al. 2015. PMID: 26426124 | |  |
| Cost of meds - GP/NP | €116.09 | Uniform*^***^* | National Health Care Institute [Zorginstituut Nederland]. Farmacotherapeutisch kompas. Available:  <http://www.farmacotherapeutischkompas.nl/>  Holtzer-Goor et al. 2015. PMID: 26426124 | |  |
| Cost of other (treatment of infections) - GP/NP | €2.53 | Uniform*^***^* |  |  |  |
| ***PT*** | | | | | |
| Consultation cost PT | €39.57 | Uniform*^***^* | Hakkaart-van Roijen L, Tan SS, Bouwmans CAM. (2011) Handleiding voor kostenonderzoek, methoden  en standaard kostprijzen voor economische evaluaties in de gezondheidszorg. geactualiseerde  versie 2010. Diemen: Department FIA CVZ.127p. | |  |
| Cost of training - PT | €207.74 | Uniform*^***^* | Expert opinion: Average of 9 - 12 therapy sessions. For the base case, 10.5 over 6 months is assumed. Upper limit is based on 12 sessions over 6 months and lower limit on 9 sessions.  Holtzer-Goor et al. 2015. PMID: 26426124 | |  |
| Cost of training plus biofeedback/electro stimulation - PT | €218.24 | Uniform*^***^* | Expert opinion: Addition of 21 euros in total (spread over 2 cycles is 10,50 per cycle).  Holtzer-Goor et al. 2015. PMID: 26426124 | |  |
| ***2nd line treatment*** | | | | | |
| Consultation cost - specialist | €131.90 | Uniform*^***^* | Dutch Healthcare Authority [Nederlandse Zorgautoriteit (NZa)] Tariffs.  Available: http://www.nza.nl/regelgeving/tarieven/ | |  |
| Cost surgery - specialist | €321.07 | Uniform*^***^* |  |  |  |
| Cost of meds - specialist | €320.26 | Uniform*^***^* |  |  |  |
| Cost of conservative therapy - specialist | €139.08 | Uniform*^***^* |  |  |  |
| ***Other direct costs inside the health care sector*** | | | | | |
| **Cost of containment pads** | | | | | |
| Cost of containment pads in improved cases | €71.92 | Uniform*^***^* | Reimbursement price. Achmea ZV. 0.78 cents per day.  Apotheek kennisbank. Available: http://www.apotheekkennisbank.nl/bedrijfsvoering/declaraties/  dagprijzen-incontinentie.  Holtzer-Goor et al. 2015. PMID: 26426124 | |  |
| Cost of containment pads in failure cases | €98.66 | Uniform*^***^* | Reimbursement price. Achmea ZV. 1.07 cents per day.  Apotheek kennisbank. Available: http://www.apotheekkennisbank.nl/bedrijfsvoering/declaraties/  dagprijzen-incontinentie.  Holtzer-Goor et al. 2015. PMID: 26426124 | |  |
| Cost of treating UTI | €2.53 | Uniform*^***^* | Hakkaart-van Roijen L, Tan SS, Bouwmans CAM. (2011) Handleiding voor kostenonderzoek, methoden  en standaard kostprijzen voor economische evaluaties in de gezondheidszorg. geactualiseerde  versie 2010. Diemen: Department FIA CVZ.127p. | |  |
| Cost of surgery for fractures | €2,954 | Uniform*^***^* | Meerding et al. 1999. PMID: 16476683  Sum of treating cost of wrist and hip fractures. | |  |
| Cost of skin breakdown | €6.55 | Uniform*^***^* | Market price of Sudocrem | |  |
| ***Direct costs outside of the healthcare sector*** | | | | | |
| **Travel costs** | | | | | |
| Travel costs to GP | €3.54 | Uniform*^***^* | Hakkaart-van Roijen L, Tan SS, Bouwmans CAM. (2011) Handleiding voor kostenonderzoek, methoden  en standaard kostprijzen voor economische evaluaties in de gezondheidszorg. geactualiseerde  versie 2010. Diemen: Department FIA CVZ.127p.  Assume 1 visit per 3 month cycle. | |  |
| Travel costs of PT | €14.08 | Uniform*^***^* | Hakkaart-van Roijen L, Tan SS, Bouwmans CAM. (2011) Handleiding voor kostenonderzoek, methoden  en standaard kostprijzen voor economische evaluaties in de gezondheidszorg. geactualiseerde  versie 2010. Diemen: Department FIA CVZ.127p.  Assume 5.25 visit per cycle. | |  |
| Travel costs to Specialist | €4.84 | Uniform*^***^* | Hakkaart-van Roijen L, Tan SS, Bouwmans CAM. (2011) Handleiding voor kostenonderzoek, methoden  en standaard kostprijzen voor economische evaluaties in de gezondheidszorg. geactualiseerde  versie 2010. Diemen: Department FIA CVZ.127p.  Assume 1 visit per cycle. | |  |
| ***Out-of-pocket costs*** | | | | | |
| Cost of containment pads for never detected but incontinent and self-care patients | €98.66 | Uniform*^***^* | Reimbursement price. Achmea ZV.  Apotheek kennisbank. Available: http://www.apotheekkennisbank.nl/bedrijfsvoering/declaraties/  dagprijzen-incontinentie.  Holtzer-Goor et al. 2015. PMID: 26426124  Assume all patients in this group are failures. | |  |
| Implementation cost of new care per patient per year | €1.51 | Uniform*^***^* | Holtzer-Goor et al. 2015. PMID: 26426124 | |  |

^*^ An standard error (SE) assuming 20% deviation from the base case value was assumed. Based on this SE, the parameters of a Beta distribution were calculated. ^**^ An SE = 0.05 was assumed. Based on this SE, the parameters of a Beta distribution were calculated. ^***^ For the Uniform distributions for the costs a 20% deviation from the base case value was assumed.
